# Cre recombinase expression cooperates with homozygous FLT3 internal tandem duplication knockin mouse model to induce acute myeloid leukemia

**DOI:** 10.1038/s41375-023-01832-0

**Published:** 2023-02-04

**Authors:** Jasmin Straube, Theresa Eifert, Therese Vu, Yashaswini Janardhanan, Rohit Haldar, Björn von Eyss, Leanne Cooper, Claudia Bruedigam, Victoria Y. Ling, Emily Cooper, Ann-Marie Patch, Lars Bullinger, Tina M. Schnoeder, Megan Bywater, Florian H. Heidel, Steven W. Lane

**Affiliations:** 1 QIMR Berghofer Medical Research Institute, Brisbane, QLD Australia; 2 The University of Queensland, Brisbane, QLD Australia; 3 Universitätsmedizin Greifswald, Innere Medizin C, Greifswald, Germany; 4 Department of Pediatrics, Section Hematology/Oncology/BMT, University of Colorado, Denver/Anschutz Medical Campus, Aurora, CO USA; 5 Leibniz Institute on Aging, Jena, Germany; 6 Hematology, Oncology and Tumor Immunology, Charite University Medicine, Berlin, Germany; 7 Royal Brisbane and Women’s Hospital, Herston, QLD Australia

**Keywords:** Acute myeloid leukaemia, Haematopoietic stem cells, Cancer models, Oncogenes

## Abstract

Murine models offer a valuable tool to recapitulate genetically defined subtypes of AML, and to assess the potential of compound mutations and clonal evolution during disease progression. This is of particular importance for difficult to treat leukemias such as FLT3 internal tandem duplication (ITD) positive AML. While conditional gene targeting by Cre recombinase is a powerful technology that has revolutionized biomedical research, consequences of Cre expression such as lack of fidelity, toxicity or off-target effects need to be taken into consideration. We report on a transgenic murine model of FLT3-ITD induced disease, where Cre recombinase expression alone, and in the absence of a conditional allele, gives rise to an aggressive leukemia phenotype. Here, expression of various Cre recombinases leads to polyclonal expansion of FLT3^ITD/ITD^ progenitor cells, induction of a differentiation block and activation of Myc-dependent gene expression programs. Our report is intended to alert the scientific community of potential risks associated with using this specific mouse model and of unexpected effects of Cre expression when investigating cooperative oncogenic mutations in murine models of cancer.

## Introduction

Mutations in FMS-like tyrosine kinase 3 (FLT3) are among the most common in myeloid blood cancers. Specifically, FLT3 internal tandem duplications (FLT3^ITD^) are found in 30–40% of all patients with acute myeloid leukemia (AML) and are associated with poor clinical prognosis due to relapse after chemotherapy [1]. Inhibitors of FLT3 combine with chemotherapy to improve overall survival in AML [2], however many patients may become resistant to these targeted inhibitors [3]. Constitutive murine models of FLT3^ITD^ have been developed that express FLT3^ITD^ from the endogenous locus [4, 5], however these mice only develop AML when crossed with another oncogenic stimulus [6–11]. These combinatorial models of leukemogenesis support a hypothesis where an oncogene driving proliferation (such as a constitutively active tyrosine kinase) can cooperate with an oncogene that blocks differentiation (e.g. the loss of function in a hematopoietic transcription factor) to drive overt AML. In light of recent discussions on the significance of FLT3^ITD^ levels on AML prognosis [12, 13], we have crossed mice heterozygous and homozygous for the FLT3^ITD^ allele with Cre recombinase expressing strains. To our surprise, we found that homozygous FLT3^ITD^ mice, when crossed with strains expressing Cre alone, had excessive and early mortality. Here we describe an unexpected but important phenomenon where Cre expression alone is able to drive AML in the context of FLT3^ITD/ITD^, but not in the Cre:FLT3^WT/WT^ or Cre:FLT3^ITD/WT^ state. This process is driven by a block in myeloid differentiation and mediated by aberrant Myc-driven transcription.

## Material and methods

### Mouse models

FLT3^ITD/ITD^ mice were obtained from Dr D. Gary Gilliland (Harvard Medical School, Boston, MA, USA) [5]. Scl^CreERT/+^ (Scl-CreERT) mice were obtained under a materials transfer agreement from Dr Joachim Göthert (University of Essen, Germany) [14]. Mx1-Cre (Strain 03556), LysM-Cre (Strain 04781), R26-LSL-Confetti (Confetti^LSL^, Strain 013731) and R26-CreER (Strain 004847) mice were obtained from Jackson Laboratories. C57BL/6J mice (6–8 weeks old) were purchased from the Animal Resources Centre (Western Australia, Australia) or from Janvier Labs (Le Genest-Saint-Isle, France) and housed in a pathogen-free animal facility. All experiments were conducted after approval by the QIMR Berghofer Animal Ethics Committee (A11605M), the Landesverwaltungsamt Sachsen-Anhalt (42502-2-1052 UniMD) and the TLV Thüringen (02-035/16). For transplantation, mice were irradiated as indicated and transplanted via tail intravenous (IV) injection with 1 × 10^5^ to 10^6^ bone marrow (BM) or spleen as indicated. Mice were culled at a defined time-point or when mice were showing significant clinical signs of disease. Disease burden was assessed by blood counts, flow cytometry (GFP+) of peripheral blood (PB), BM and spleen cells or histopathological stains.

#### Cre activation

To activate Cre in the Scl-CreERT and R26-CreER models, tamoxifen feed purchased from Specialty Feeds (Western Australia) at 400 mg/kg in base Mouse FG 66305 formulation was used. To activate Mx1-Cre in vivo, poly(I):poly(C) (pIpC) (Cytiva, Marlborough, MA) was injected two times every second day intraperitoneally. Injections were halted if mice showed signs of illness prior to completion of treatment. Spontaneous Mx1-Cre activation was noted as previously described consistent with spontaneous activation of Mx1-Cre in an inflammatory milieu [15].

#### In vivo treatment with JQ1

Scl-CreERT:FLT3^ITD/ITD^ or age-matched FLT3^ITD/ITD^ mice were administered tamoxifen feed (Specialty Feeds Australia) for 4 weeks, then blood was sampled and analysed on a Hemavet 950 (Drew Scientific) to confirm leukocytosis. Tamoxifen feed was withdrawn for 4 days prior to JQ1 treatment. JQ1 was formulated by dissolving 100 mg/mL in DMSO (1 part) and diluting by dropwise addition of 10% hydoxypropyl beta-cyclodextrin (Sigma Aldrich, 332593) (9 parts), followed by sonication for 15 min in a water bath sonicator at 37 °C. Mice were injected intraperitoneally at 50 mg/kg of JQ1 or vehicle, for 2 weeks (5 days per week). Mouse tissue samples were harvested after day 12 of JQ1 treatment and immunophenotyped using the LSRII Fortessa analyzer (BD). For samples used for RNA-sequencing, lethally irradiated (1100 cGy) Ptprca mice were transplanted with Scl-CreERT:FLT3^ITD/ITD^ leukemic splenocytes (5 × 10^6^) mixed with C57BL/6 BM (5 × 10^5^). AML was confirmed after 4 weeks and mice were treated with one dose of JQ1 (50 mg/kg) or vehicle (200 uL of 10% cyclodextrin/DMSO). After 4 h, spleens were harvested sorted for Kit+CD45.2 + CD45.1- cells and RNA was extracted using the PicoPure RNA Isolation kit (Thermo Fisher).

Sample sizes for animal experiments were calculated to detect 25% difference in disease burden parameters between groups at a power of 80% with alpha of 0.05. Treatment groups were pre-specified prior to transplantation and treatment allocation was non-randomized and non-blinded throughout the experiment. All mice were included in analyses.

### Blood analysis and bone marrow cytospins

Blood was collected into EDTA-coated tubes and blood counts assessed using a Hemavet 950 analyzer (Drew Scientific) or on a BC-5000Vet (Mindray, China). To analyse cell morphology, 1 × 10^5^ BM cells were centrifuged onto glass slides. PB smears and BM cytospins were stained with Wright-Giemsa (BioScientific).

### Histological imaging of mouse organs

Spleen, liver and lung were fixed and embedded according to standard protocols. Slides were automatically processed for hematoxylin and eosin staining (Leica AutoStainer XL, Leica Biosystems, Wetzlar, Germany). Images were acquired at 10x magnification on an AxioImager A.2 (Carl Zeiss Microscopy, Jena, Germany). Images were processed and analysed using the ZEN software (blue edition, version 2.3, Carl Zeiss Microscopy GmbH, Jena, Germany).

### Flow cytometry

For immunophenotype analysis, PB, BM or spleen cells were resuspended in PBS/1% FBS after erythrocyte lysis (PharmLyse^TM^, BD Pharmingen, San Diego, CA). Unless otherwise stated, the following antibodies were used: Sorting and analysis of Lineage^-^ Kit+ Sca-1 + (LKS+) cells or Sca-1+ cells were performed as previously described [16, 17]. Biotinylated antibodies against Gr-1 (RB6-8C5), B220 (RA3-6B2), CD19 (6D5), CD3 (145-2C11), CD4 (GK1.5), CD8a (53–6.7), TER119 and IL7Ra (A7R34) (all Biolegend, SanDiego, CA) were used for lineage staining. An APC-Cy7- or BV421-labeled streptavidin-antibody (BioLegend) was used for secondary staining together with an APC-anti-cKit (clone 2B8) and a PE-Cy7- or PE-anti-Sca-1 antibody (clone E13-161.7). Full list of antibodies can be found in Supplementary Table 1. Cells were analysed using an BD-Fortessa, LSRII^TM^ or FACSCantoII^TM^ (Becton-Dickinson) cytometer. Analysis was performed using FlowJo^TM^ software (Treestar, Ashland, OR). Cell sorting was performed on a BD FACSAria™ II (Becton-Dickinson).

### Molecular protocols and next-generation sequencing

#### DNA extraction

Isolation of gDNA from Mx1-Cre:FLT3^ITD/ITD^ AML BM cells or FLT3^WT/WT^ tail biopsies was performed using the QIAmp DNA Blood Mini Kit or the QIAmp Fast DNA Tissue Kit (Qiagen, Hilden, Germany), respectively, according to the manufacturer’s instruction. Genomic DNA was extracted using QIAGEN QIAquick from Cre+FLT3^ITD/ITD^ tail pre-tamoxifen (control) and BM post-tamoxifen at clinical signs of significant disease (tumor).

#### Whole exome sequencing (WES)

100 bp paired end sequencing was performed on the HiSeq4000 Illumina platform with 30–40x coverage through Macrogen. Variants were called and annotated using qSNP [18].

#### Whole Genome sequencing (WGS)

Cre+FLT3^ITD/ITD^ tail pre-tamoxifen and BM post-tamoxifen were sequenced through Macrogen with 150 bp paired end on the Illumina platform at 22x and 38x coverage, respectively. WGS of WT tails and BM of Mx1-Cre:FLT3^ITD/ITD^ pre-pIpC were performed by Genewiz (Leipzig, Germany) on Illumina NovaSeq 150 bp paired end 30x coverage.

#### Flt3 Neomycin resistance cassette sequence assembly

WGS reads mapping to intron between Flt3 Exon15 and Exon 16 and reads not mapped to the mouse genome were extracted from WGS of Cre+FLT3^ITD/ITD^ pre tamoxifen mice and de novo assembled using velvet v1.2.10. Primers were designed adjacent to Neo-r-cassette integration site targeting the Flt3 locus: Flt3 I15 F1 5′ GCAATGTCAGAACACGATCACT 3′, Flt3 I15 R2 5′ CAGGAGATGAAGCTGGGTTATAG. Sanger sequencing was performed with primer pairs Flt3 neo Seq F1 5′ GAATATGATCGGAATTCCTCG 3′, Flt3 neoSeq R1 5′ CAGGTCGAGCAGTGTGGTT 3′ and Flt3 neo Seq F2 5′ GATCCGAACAAACGACCCAAC 3′, Flt3 neoSeq R2 5′ TACGTCCAGCCAAGCTAGC 3′, to confirm the in silico generated Neo-r-cassette reference sequence and annotated through blast. WGS reads of tails from WT, pre-taxmoxifen Cre+FLT3^ITD/ITD^ and BM post-tamoxifen Cre+FLT3^ITD/ITD^ were mapped against the Neo-r-cassette reference using bwa mem.

#### RNA extraction, sequencing and analysis

For Scl-CreERT:FLT3^ITD/ITD^ and age-matched FLT3^ITD/ITD^ control BM, LKS + at 4 weeks post tamoxifen and spleen Lineage^-^ Kit + (LK) at significant clinical signs of disease were sorted and RNA isolated using Arcturus PicoPure RNA Isolation Kit (Thermo Fisher Scientific, Waltham, MA). For Mx1-Cre:FLT3^ITD/ITD^ and control BM, 1 × 10^4^ to 2 × 10^5^ LKS + cells were sorted 10 days after last pIpC treatment into TRIzol® (Thermo Fisher Scientific, Waltham, MA) and RNA was isolated according to the manufacturer’s instruction. RNA libraries were prepared using the NEBnext Ultra RNA Library Prep Kit for Illumina (New England Biolabs), assessed for size and quantified using the 2100 Bioanalyzer (Agilent Technologies) and Kapa Library Quantitation Kit (Illumina) respectively, prior to sequencing on the Illumina NextSeq 500 platform (75 bp single end). Reads were adapter trimmed (Cutadapt [19] v1.11) and aligned (STAR [20] v2.5.2a) to the GRCm38 assembly using the gene, transcript, and exon features of Ensembl (release 70) gene model. Expected gene counts were estimated with RSEM [21] v1.2.30. Genes with zero read counts across all samples were removed prior analysis. Reads were normalisation using edgeR [22] (counts per million, CPM; trimmed mean of M-values; TMM) and used for gene set enrichment analysis (GSEA) analysis [23]. Differential expression analysis comparing Cre+FLT3^ITD/ITD^ vs. FLT3^ITD/ITD^ was performed with edgeR using a negative binomial generalised log-linear model paired with genewise likelihood ratio tests.

#### ATACSeq sample processing and analysis

BM LKS+ from Scl-CreERT:FLT3^ITD/ITD^ mice were FACS sorted at Scl-CreERT:FLT3^ITD/ITD^ significant signs of disease along with aged-matched FLT3^ITD/ITD^ control LKS+. Cells were washed in ice cold PBS, pelleted and lysed in 50ul of lysis buffer (10 mM Tris-HCl pH 7.4, 10 mM NaCl, 3 mM MgCl2, 0.1% (v/v) NP40). The lysate was centrifuged at 500 *g* for 10 mins. DNA tagmentation and library prep were performed on the nuclear pellet using the Nextera DNA Library Prep Kit (Illumina). The nuclei were resuspended in 25ul tagmentation buffer with 22.5uL H_2_0 and 2.5uL transposase and incubated at 37 °C for 30 min. Tagmented chromatin was purified using a MinElute PCR Purification Kit (Qiagen). Libraries added 2.5uL of both the forward and reverse indexing primers (25uM) to tagmented DNA and 25uL of KAPA HiFi HotStart ReadyMix (KAPABiosystems Millenium). Tagmented chromatin was amplified by PCR for 72 °C 5 min, 98 °C 3 mins, followed by 13 cycles at 98 °C 20 s, 65 °C 15 s, 72 °C 60 s, purified using a MiniElute PCR Purification Kit (Qiagen), eluted in 50uL prior to SPRI based size selection (200–600 bp) (Beckman Coulter), Qubit flurometric quantitation and sizing and DNA analysis using a Bioanalyzer (2100 Agilent). Sequencing was performed by Macroogen with 100 bp paired end on the Illumina platform. Reads were trimmed for Nextera transposase adapter sequences (Cutadapt v1.11) and aligned (bwa mem v0.7.15) to mm9 genome. Mapped reads were assessed for duplication with picard v2.18.15 MarkDuplicates and filtered in addition to low mapping quality (q < 30) using samtools v1.9. Peaks were called with MACS2 v2.1 ‘macs2 callpeak -t output.bam --format --gsize mm --nomodel --nolambda --keep-dup all --call-summits’. Peaks overlapping blacklisted regions were removed using GenomicRanges v1.48. Peaks were annotated according to their location with ChIPseeker v1.32. Differential peak analysis was performed using edgeR as previously described. Caterpillar plots were generated with genomation R package v1.28. ATACSeq data of murine HSPC and mature cell populations were downloaded from GEO with accession number GSE60103.

### Statistical Analysis

Kaplan-Meier curves were plotted using GraphPad Prism version 9.0 (GraphPad Software, San Diego, CA) using the log-rank test (Mantel-Cox test). Statistical analyses were performed using ANOVA with FDR p-value correction for comparing more than two groups or t-test for comparing two groups, unless stated otherwise. Significance of p-values in figures are indicated using the following ranges: * *p* < 0.05; ** *p* < 0.01; *** *p* < 0.001; **** *p* < 0.0001. Each dot represents an individual biological replicate.

## Results

### Expression of Cre recombinase cooperates with FLT3^ITD^ to induce acute myeloid leukemia (AML) in mice

**Fig. 1:**
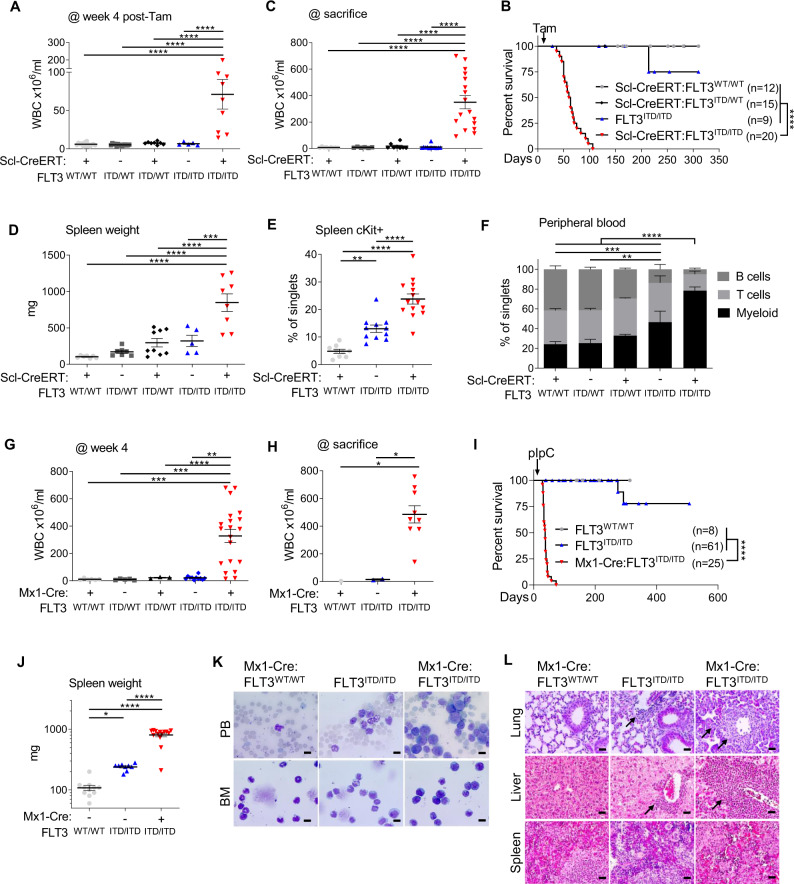
Cre recombinase cooperates with FLT3^ITD/ITD^ to induce lethal AML. Genotypes represented in **A-F** are Scl-CreERT:FLT3^WT/WT^, FLT3^ITD/WT^ Scl-CreERT:FLT3^ITD/WT^, FLT3^ITD/ITD^ and Scl-CreERT:FLT3^ITD/ITD^. **A** Peripheral blood white cell count (WBC) 4 weeks after tamoxifen treatment. Each point represents an individual mouse. **B** WBC at termination of experiment. **C** Kaplan-Meyer survival curves of Scl-CreERT:FLT3^WT/WT^ (*n* = 12), Scl-CreERT:FLT3^ITD/WT^ (*n* = 15), FLT3^ITD/ITD^ (*n* = 9) and Scl-CreERT:FLT3^ITD/ITD^ (*n* = 20), **D** Spleen weight **E** Immunophenotyping of Kit+ spleen cells **F** Immunophenotyping of peripheral blood cells by flow cytometry (CD11b + myeloid cells, CD19 + B cells, CD3 + T cells). **G-K** Genotypes are Mx1-Cre:FLT3^WT/WT^, FLT3^ITD/WT^ Mx1-Cre:FLT3^ITD/WT^, FLT3^ITD/ITD^ and Mx1-Cre:FLT3^ITD/ITD^. **G** WBC at 4 weeks and **H** at termination of experiment. **I** Kaplan-Meyer survival curves of Mx1-Cre:FLT3^WT/WT^ (*n* = 8), FLT3^ITD/ITD^ (*n* = 61) and Mx1-Cre:FLT3^ITD/ITD^ (*n* = 25), **J** Spleen weight and **K** morphology of leukocytes in the peripheral blood and bone marrow, demonstrating loss of differentiation in Mx1-Cre:FLT3^ITD/ITD^ (upper panel). **L** Histopathology (HE-staining) of organs from Mx1-Cre:FLT3^WT/WT^, FLT3^ITD/ITD^ and Mx1-Cre:FLT3^ITD/ITD^ mice (lower panel). Statistics: **A**-**E**, **G**, **H**, **J** One-way ANOVA with FDR p-value correction, **C**, **I** Pairwise Mantel-Cox test for survival analysis, **F** two-way ANOVA with Tukey p-value correction; p-values from comparing myeloid cells displayed.

FLT3^ITD^ is the most common recurrent genetic mutation found in patients with AML. FLT3^ITD/ITD^ knockin mice have been a valuable research model for studying the effect of cooperative gene mutations because they only develop a chronic myeloproliferative disease. Recently, other groups have described development of AML in the presence of additional mutations [6]. Scl-CreERT expression is restricted to hematopoietic stem and progenitor cells (HSPC) [14]. Scl-CreERT, Scl-CreERT:FLT3^ITD/WT^, FLT3^ITD/ITD^ and Scl-CreERT:FLT3^ITD/ITD^ mice were generated and born in expected Mendelian ratios. At 6–8 weeks after birth, mice were treated with tamoxifen-supplemented chow to induce the activity of Cre recombinase. Unexpectedly, Scl-CreERT:FLT3^ITD/ITD^ mice developed signs of sickness within 4 weeks including marked leukocytosis (Fig. 1A, B). This progressed to a lethal AML characterised by splenomegaly and increased Kit+ HSPC in the spleen, progressive anaemia and marked myeloid skewing in the peripheral blood (PB) (Fig. 1C-F, Fig. S1A). These findings were not present in the Scl-CreERT control groups and significantly greater than in the Scl-CreERT:FLT3^ITD/WT^ or FLT3^ITD/ITD^ control groups. Notably, FLT3^ITD/ITD^ control groups also showed myeloid skewing and splenomegaly, consistent with the previous publications [5]. Phenotypically immature blasts were detectable in PB and BM, consistent with AML (Fig. S1B). To determine whether this effect was present with other Cre recombinases, we crossed FLT3^WT/WT^, FLT3^ITD/WT^ and FLT3^ITD/ITD^ mice with Mx1-Cre, an interferon inducible Cre recombinase transgene with high expression in hematopoietic cells [24]. Mx1-Cre:FLT3^WT/WT^, Mx1-Cre:FLT3^ITD/WT^, and Mx1-Cre:FLT3^ITD/ITD^ mice were initially treated with pIpC at 4 weeks after birth. Consistent with our previous findings, Mx1-Cre:FLT3^ITD/ITD^, developed leukocytosis and this progressed to a rapidly fatal AML accompanied by splenomegaly and immature blasts in PB and BM whereas control mice did not develop AML (Fig. 1G-K). Of note, disease development was detectable in a subset of mice without pIpC injection due to low level expression from the Mx1 promoter in the absence of an ectopic stimulus. Together, these unexpected findings show that Cre expression alone is sufficient to cooperate with FLT3^ITD/ITD^ to induce AML in mouse models.

### Cre + FLT3^ITD/ITD^ AML is transplantable and maintained by transformed HSPC populations

**Fig. 2:**
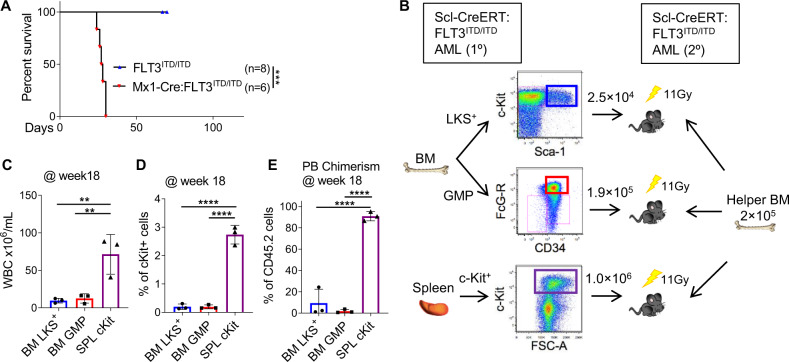
Cre:FLT3^ITD/ITD^ AML is transplantable in recipient mice. **A** Kaplan-Meyer survival curves of recipient mice. Injection of Mx1-Cre:FLT3^ITD/ITD^ vs. FLT3^ITD/ITD^ bone marrow cells into irradiated C57BL/6 J recipient hosts (Mx1-Cre:FLT3^ITD/ITD^, *n* = 6; FLT3^ITD/ITD^, *n* = 8). **B** Schema of secondary transplantation experiments from Scl-CreERT:FLT3^ITD/ITD^ cells derived from BM (sorted LKS + or GMP) vs. spleen (Kit + ) into lethally irradiated C57BL/6 J recipient together with 2 × 10^5^ helper BM cells. **C** Elevated WBC **D** expanded Kit+ population and **E** engraftment of CD45.2 positive AML cells enriched in recipients of spleen Kit+ cells. Each dot represents an individual biological replicate (mouse). Statistics: **A** Pairwise Mantel-Cox test, **C-E** One-way ANOVA with FDR p-value correction.

To determine whether Cre+FLT3^ITD/ITD^ AML is fully transformed, we performed transplantation into lethally irradiated (1100 cGy) C57BL/6 J recipient mice. Whole BM of Mx1-Cre:FLT3^ITD/ITD^ or Scl-CreERT:FLT3^ITD/ITD^ but not FLT3^ITD/ITD^ was able to transplant a short latency, fatal AML into recipient mice (Fig. 2A). Next, we performed fractionation by flow cytometry, isolating putative leukemia initiating cell populations (LKS + , GMP) from the BM and spleen (Kit + ) of leukemic mice and transplanted these cells along with 2 × 10^5^ wildtype BM cells (“helper BM”) into lethally (1100 cGy) irradiated mice (Fig. 2B). AML was able to be transplanted in recipient mice that received 1 × 10^6^ Kit+ cells from AML spleens (Fig. 2C, D). Additionally, BM LKS + cells were able to engraft into irradiated recipient, but did not manifest disease, presumably due to the limiting cell number used in transplantation (Fig. 2E). However, these results show that Cre+FLT3^ITD/ITD^ AML can be propagated in irradiated recipient mice and that the HSPC population contains AML initiating activity in vivo. We therefore sought to characterise the effect of Cre recombinase on HSPC populations.

### Cre + FLT3^ITD/ITD^ AML leads to expansion of committed HSPC populations with leukemia initiating activity

**Fig. 3:**
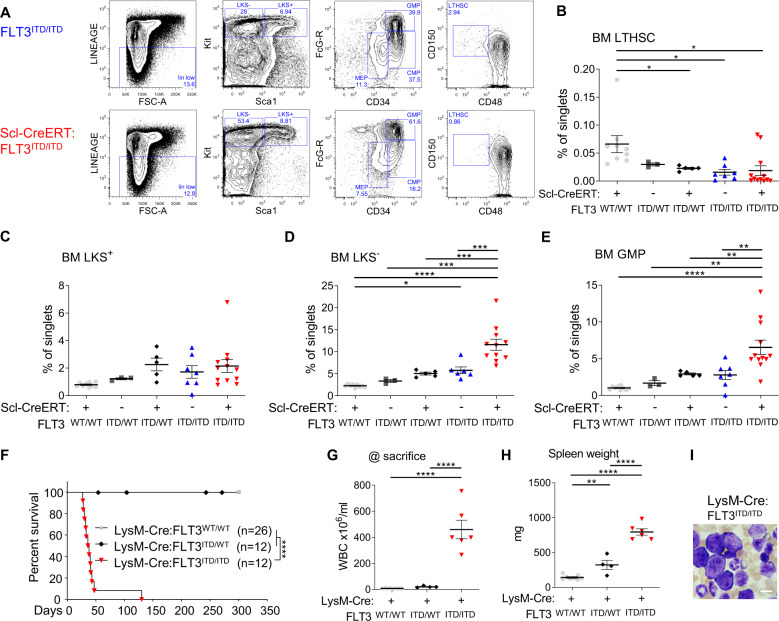
Cre + FLT3^ITD/ITD^ leads to expansion of HSPC populations within the bone marrow. Genotypes represented in **A-E** are Scl-CreERT:FLT3^WT/WT^, FLT3^ITD/WT^ Scl-CreERT:FLT3^ITD/WT^, FLT3^ITD/ITD^ and Scl-CreERT:FLT3^ITD/ITD^. **A** FACS plots showing expansion of mixed myeloid progenitors (LK + Sca-1-) in Scl-CreERT:FLT3^ITD/ITD^. **B** Long-term (LT) HSCs (LKS + CD150 + CD48-), **C** BM LKS + cells, **D** BM LKS- cells and **E** Granulocyte macrophage progenitors (GMP). **F-I** Genotypes represented are LysM-Cre:FLT3^WT/WT^, LysM-Cre:FLT3^ITD/WT^, and LysM-Cre:FLT3^ITD/ITD^. **F** Kaplan-Meyer survival curves of LysM-Cre:FLT3^WT/WT^ (n = 26), LysM-Cre:FLT3^ITD/WT^ (n = 12), and LysM-Cre: FLT3^ITD/ITD^ (n = 12) **G** WBC and **H** spleen weight at termination of experiment. **I** Peripheral blood blast morphology in LysM-Cre:FLT3^ITD/ITD^. Statistics: **B**-**E**, **G**, **H** One-way ANOVA with FDR p-value correction, **F** pairwise Mantel-Cox test.

Consistent with the aggressive disease phenotype, AML-bearing Scl-CreERT:FLT3^ITD/ITD^ mice had the fewest immunophenotypically defined long-term HSCs, however this was not significantly different between the Cre+ and Cre- FLT3^ITD/ITD^ controls (Fig. 3A, B). AML mice from Scl-CreERT:FLT3^ITD/ITD^ showed marked expansion of BM HSPC populations, particularly in the myeloid progenitor compartment (Lineage^-^ Kit+ Sca-1^-^), beyond that observed with FLT3^ITD/ITD^ alone (Fig. 3C-E). More specifically, AML-bearing Scl-CreERT:FLT3^ITD/ITD^ mice had expansion of cells expressing markers consistent with granulocyte macrophage progenitors (GMP) (Fig. 3E). As these data suggest that Cre induction preferentially leads to expansion of a committed myeloid HSPC population, we examined the effect of Cre expression within the GMP and committed myeloid progenitor compartment using LysM-Cre, a constitutive Cre within myeloid cells that has maximal expression from GMP stage but may be active in a small number of HSCs [25]. Consistent with the previous findings, LysM-Cre:FLT3^ITD/ITD^ mice also developed a rapidly fatal AML (Fig. 3F) characterised by extremely high WBC count, splenomegaly and circulating blast cells (Fig. 3G-I).

### Cre + FLT3^ITD/ITD^ AML is polyclonal and not associated with recurrent genetic mutations

**Fig. 4:**
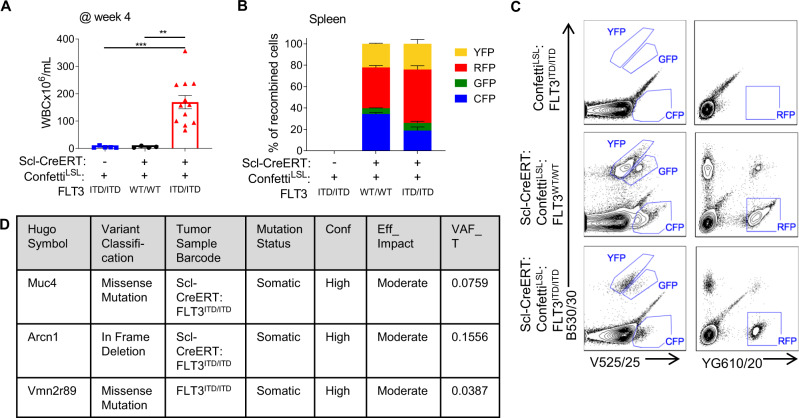
Cre + FLT3^ITD/ITD^ AML is polyclonal and not associated with recurrent genetic driver lesions. **A** Genotypes represented in **A-D** are Scl-CreERT:Confetti^LSL^, Confetti^LSL^:FLT3^ITD/ITD^ and Scl-CreERT:Confetti^LSL^:FLT3^ITD/ITD^. **A** WBC at 4 weeks after induction of Cre with tamoxifen. **B** Percentage of Confetti marked cells that express either Yellow, Red, Green or Cyan Fluorescent Protein. **C** Representative flow cytometry plots corresponding with **B**. **D** Table of somatic mutations found when comparing exome sequencing in FLT3^ITD/ITD^ and Scl-CreERT:FLT3^ITD/ITD^. **A** One-way ANOVA with FDR p-value correction.

We next sought to determine whether Cre recombinase was driving the leukemia phenotype through the generation of additional oncogenic mutations. We first tested whether the leukemic phenotype was associated by a dominant clonal population that expanded and gave rise to AML by generating Scl-CreERT:Confetti^LSL^:FLT3^ITD/ITD^ and Scl-CreERT:Confetti^LSL^:FLT3^WT/WT^ mice that would randomly express either GFP, YFP, CFP or RFP upon the induction of Cre recombinase, allowing us to trace clonal evolution based on fluorescent marker expression. After tamoxifen induction of Cre recombinase in HSCs, only Scl-CreERT:Confetti^LSL^:FLT3^ITD/ITD^ mice developed AML (Fig. 4A). Although there was incomplete expression of each fluorescent marker, we observed that the ratios of each fluorochrome were similar between both conditions, demonstrating that the AML was polyclonal in Scl-CreERT:Confetti^LSL^:FLT3^ITD/ITD^ (Fig. 4B, C). Next, we examined whole exome sequencing (WES) of Scl-CreERT:FLT3^ITD/ITD^ AML cells after tamoxifen administration to determine whether recurrent genetic mutations were found in the AML cells. WES of cells isolated from AML-bearing BM was compared to tail gDNA prior to Cre induction and mutations defined as somatic if present in the BM but not in the tail samples. Somatic mutations were filtered based on high confidence calls with moderate or high predicted impact on protein function. Scl-CreERT:FLT3^ITD/ITD^ AML had only two somatic mutations, in the genes *Muc4* and *Arcn1*, with low variant allele frequency (VAF 0.075 and 0.155, respectively), whereas FLT3^ITD/ITD^ controls had a sole mutation in *Vmn2r89* (low VAF, 0.039) (Fig. 4D; Supplementary Table 2). As these mutations were found in a minority of cells, we concluded that they were unlikely to be additional driver mutations responsible for the AML phenotype.

### FLT3^ITD/ITD^ cells have a retained Neomycin resistance cassette that is LoxP flanked and excised by Cre recombinase

**Fig. 5:**
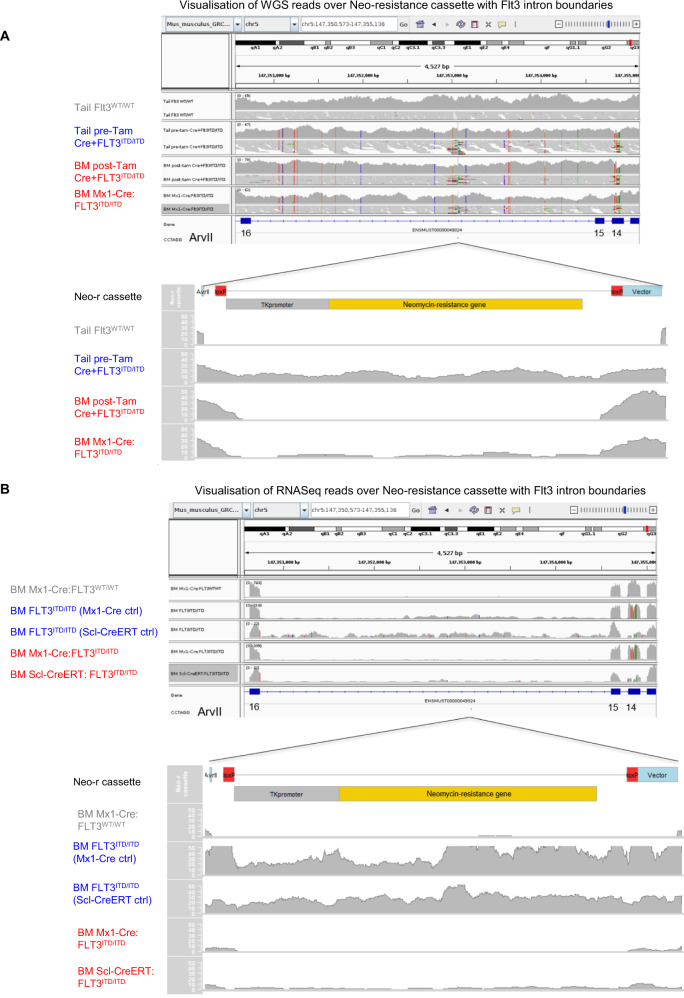
Genomic and transcriptomic analysis of the FLT3 locus. **A** Visualisation of whole genome sequencing (WGS) reads over the endogenous Flt3 intron between exon 15 and 16 (top) and the neomycin (Neo) resistance cassette with flanking Flt3 intronic sequence (bottom) in Cre+FLT3^ITD/ITD^ pre Cre expressing cells demonstrating retained targeting vector reads that map to a Neo-resistance cassette. This is not present in WT cells or after Cre expression. **B** RNA-sequencing reads over the endogenous Flt3 intron between exon 15 and 16 (top) and the Neo-resistance cassette with flanking Flt3 intronic sequence (bottom) consistently showing reads in FLT3^ITD/ITD^ cells but not in Scl-CreERT:FLT3^ITD/ITD^, Mx1-Cre:FLT3^ITD/ITD^ cells post Cre expression or Mx1-Cre:FLT3^WT/WT^.

We next performed whole genome sequencing (WGS) of pre-tamoxifen Cre+FLT3^ITD/ITD^ tails and post-tamoxifen AML BM cells and FLT3^WT/WT^ tails and pre-pIpC BM of Mx1-Cre:FLT3^ITD/ITD^ AML samples. We observed that pre-tamoxifen Cre+FLT3^ITD/ITD^ samples but not post-tamoxifen AML Cre+FLT3^ITD/ITD^ cells retained an aberrant neomycin-resistance sequence between exon 15 and 16 of *FLT3* flanked by LoxP sites, a residual gene targeting sequence inserted during generation of this mouse model [5] (Fig. 5A). Similar findings have been described from another Flt3^ITD^ knockin mouse model [4]. Consistent with this, RNA-sequencing from both FLT3^ITD/ITD^ models contained reads mapping to the neomycin-resistance cassette pre but not post Cre induction (Fig. 5B). Altogether, these data suggest that Cre recombinase deletes a retained neomycin-resistance expression cassette, with possible implications for gene regulation and gene expression.

### Cre + FLT3^ITD/ITD^ AML has aberrant gene expression leading to differentiation block and Myc activation

**Fig. 6:**
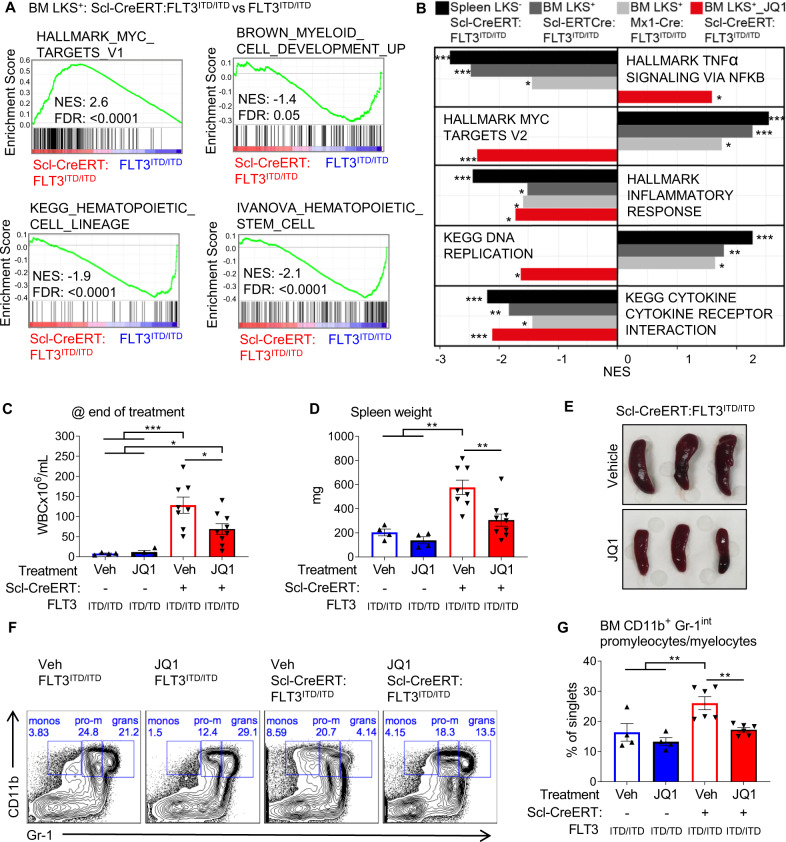
Concordant gene expression changes with each model demonstrate activation of a pro-proliferative Myc signature and loss of myeloid differentiation. **A** Gene set enrichment analysis (GSEA) from Scl-CreERT:FLT3^ITD/ITD^ vs. FLT3^ITD/ITD^ BM LKS + cells show that Cre positive cells have enrichment for Myc signature, and negative enrichment for signatures associated with myeloid differentiation and mature hematopoietic cells. **B** Concordance analysis demonstrating that the gene expression changes seen are similar between BM LKS + cells from Mx1-Cre:FLT3^ITD/ITD^, Scl-CreERT:FLT3^ITD/ITD^ and spleen Kit+ Scl-CreERT:FLT3^ITD/ITD^ cells. These changes are reversed by the BRD4 inhibitor JQ1 in vivo. **C** WBC and **D** spleen weight at the end of treatment from FLT3^ITD/ITD^ or Scl-CreERT:FLT3^ITD/ITD^ mice with or without JQ1 treatment demonstrating partial reversal of leukocytosis and splenomegaly. **E** Representative pictures of spleens after JQ1 treatment. **F** Representative flow cytometry plots and **G** quantitative analyses of bone marrow immunophenotyping demonstrating partial reversal of the myeloid differentiation block after JQ1 treatment. Statistics: **A**, **B** GSEA with FDR p-value correction; **C**, **D**, **G** One-way ANOVA with FDR p-value correction.

To determine whether Cre+FLT3^ITD/ITD^ AML was driven by broad changes in gene expression that were concordant across different Cre genotypes, we interrogated RNA-sequencing on Kit+ populations from Scl-CreERT:FLT3^ITD/ITD^ spleen and BM and Mx1-Cre:FLT3^ITD/ITD^ BM compared to FLT3^ITD/ITD^ controls. Despite being isolated from immunophenotypically similar cells, there were striking gene expression changes in both Cre-positive groups (Fig. S2A), characterised by a loss of myeloid differentiation and enrichment for stem cell regulatory pathways (Fig. 6A) with differential expression of key myeloid transcription factors (Fig. S2B) compared to FLT3^ITD/ITD^ controls. There was strong and significant concordance between the gene expression changes seen with Scl-CreERT:FLT3^ITD/ITD^ AML and Mx1-Cre:FLT3^ITD/ITD^ AML (Fig. S2C). FLT3^ITD/ITD^ mice are characterised by an aberrant inflammatory milieu and disrupted cytokine signalling, and these changes were lost in both FLT3^ITD/ITD^ Cre-positive groups (Fig. 6B, Supplementary Table 3).

We therefore considered whether Cre-activity may have an effect on remodelling chromatin or binding at specific sites of the genome to regulate these gene expression changes. We therefore performed genome wide ATACSeq on BM LKS + cells from FLT3^ITD/ITD^ samples and Scl-CreERT:FLT3^ITD/ITD^ samples to examine chromatin conformation in an unbiased manner. Chromatin accessibility was highly consistent between biological replicates of genotypes (Fig. S3A) with Scl-CreERT:FLT3^ITD/ITD^ samples overall gaining chromatin accessibility (Fig. S3B, Supplementary Table 4). Interestingly, Scl-CreERT:FLT3^ITD/ITD^ preferentially lost open chromatin at the sites accessible during myeloid differentiation with enrichment for PU.1 (encoded by Sfpi1) motif (Fig. S3C–F). We reasoned that this was consistent with the block in differentiation phenotype seen in the BM Scl-CreERT:FLT3^ITD/ITD^ samples overall and did not suggest specific binding of Cre recombinase to directly influence gene expression.

We did observe increase in protein FLT3 expression in Mx1-Cre:FLT3^ITD/ITD^ mice (Fig. S4A–K). Additionally, we see strong and consistent findings using RNA-sequencing analysis of Mx1-Cre, Scl-CreERT in BM and spleen are upregulation of known FLT3 target gene Socs1 (Fig. S4L) with consequent suppression of cytokine and inflammatory signaling (Fig. 6D) particular interferon signaling (Fig. S4L). Socs1 expression is relevant to this as since in a retroviral model it was shown to significantly accelerate the FLT3^ITD^ myeloproliferative phenotype or leads to leukemia [26].

In addition, RNA-sequencing data from both Scl-CreERT:FLT3^ITD/ITD^ AML and Mx1-Cre:FLT3^ITD/ITD^ AML showed marked enrichment for genes associated with Myc activation (Fig. 6B). To confirm that Myc activation was driving the development or maintenance of AML, we assessed response to targeting Myc with the BRD4 inhibitor JQ1 [27]. Cre-negative FLT3^ITD/ITD^ mice did not develop increased WBC count, splenomegaly or monocyte differentiation block and hence did not show any response in these parameters after 2 weeks of JQ1 treatment (Fig. 6C, D, G). Scl-CreERT:FLT3^ITD/ITD^ mice showed a marked accumulation of immature monocytes, elevated WBC and splenomegaly compared to Cre-negative controls (Fig. 6C–G). However, Scl-CreERT:FLT3^ITD/ITD^ mice treated with JQ1 showed a reduction in WBC counts, decreased splenomegaly and partial reversal of abnormal myeloid differentiation (Fig. 6C–G). Gene expression studies on JQ1 treated Scl-CreERT:FLT3^ITD/ITD^ AML showed reversal of Myc regulated gene expression and restoration of TNF***α*** signalling pathways seen upregulated in Scl-CreERT:FLT3^ITD/ITD^ mice (Fig. 6B). In aggregate, these findings demonstrate that Cre expression leads to broad gene expression changes, including abnormal Myc pathway activation leading to a block in differentiation that drives AML development in FLT3^ITD/ITD^ cells.

## Discussion

Genetically engineered mouse models faithfully recapitulate many myeloid malignancies and provide important mechanistic insights that may not be evident from studying human samples alone. The transgenic FLT3^ITD/ITD^ knockin model has been used widely to model the effects of cooperative mutations on leukemogenesis, including NPM1 [9, 10] and DNMT3A [6, 28]. We present the unexpected findings that homozygous FLT3^ITD/ITD^ mice are primed for AML development and that co-expression of Cre recombinase is sufficient to give rise to a fully penetrant, yet polyclonal AML. This AML shows many characteristics of human cancer including a differentiation block and activation of a transcriptional Myc signature. Mechanistically, Myc activation appears to be important for the phenotypic manifestations of disease, as treatment with the BRD4 inhibitor JQ1 was able to reverse these gene expression changes and partially restore differentiation in vivo. This phenotype could not be attributed to any spurious damage to the genome by Cre recombinase as we did not find evidence of gene coding mutations. However, we did identify a retained neomycin resistance cassette that was incorporated in the Flt3 locus at the time of gene targeting. This Neo-resistance cassette is intronic in the Flt3 locus, is flanked by LoxP sites and is predicted to reduce expression of FLT3 overall [4]. After Cre-induction, this Neo-resistance cassette is excised, suggesting that retention of this intronic Neo-resistance construct may be having broader impact on gene expression.

Importantly, we do not propose this model as a useful preclinical tool that faithfully mimics the progression of human disease. Rather, this is a cautionary report that serves to notify and remind the broader scientific community of the potential caveats of this particular mouse model in modelling AML, but also has impact more generally about the potential for off-target effects of gene targeting as previously emphasized by pioneers of the Cre-loxP system [29]. This finding may limit the applicability of data to clinical scenarios. This finding was only detected by the careful analysis of Cre-positive (and excision positive) controls, as Cre-negative FLT3^ITD/ITD^ mice never develop AML. Heterozygous expression of FLT3^ITD/WT^ in the presence of Cre activity did not give rise to off-target AML in any of our experiments, and we believe that this model remains suitable and appropriate for the testing of cooperative event in AML.

## Supplementary information

Supplementary Fig. S1

Supplementary Fig. S2

Supplementary Fig. S3

Supplementary Fig. S4

Supplementary Table S1

Supplementary Table S2

Supplementary Table S3

Supplementary Table S4

## Data Availability

ATAC- and RNA-sequencing data are available through NCBI Gene Expression Omnibus under accession numbers GSE212222. Whole Exome- and Whole Genome-Sequencing analysis have been deposited in the NCBI sequencing read archive, BioProject ID: PRJNA909339.
