# Supplementary figures and images for: Cre recombinase expression cooperates with homozygous FLT3 internal tandem duplication knockin mouse model to induce acute myeloid leukemia
Source: Leukemia. 2023 Feb 4;37(4):741–50. doi: 10.1038/s41375-023-01832-0 (PMC10079527; doi:10.1038/s41375-023-01832-0)

Supplementary Figure 1

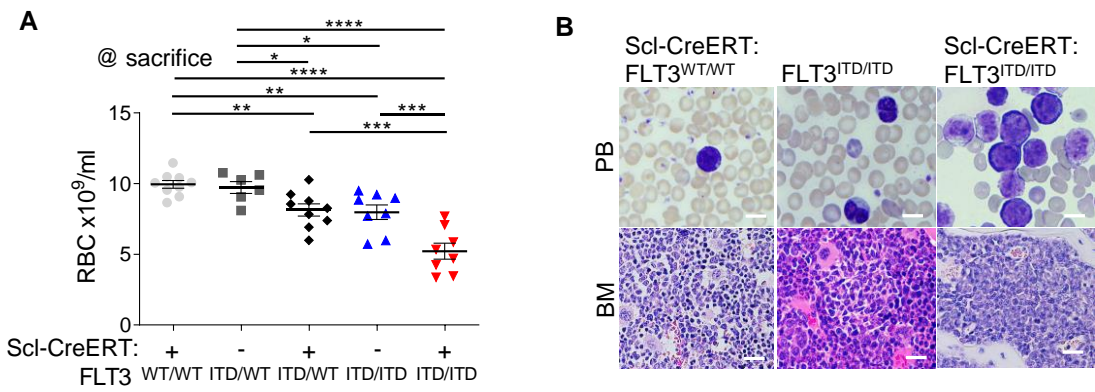

Supplement: Supplementary file 1 — Supplementary Fig. S1 [file 41375_2023_1832_MOESM1_ESM.pdf]

Supplementary Figure 2

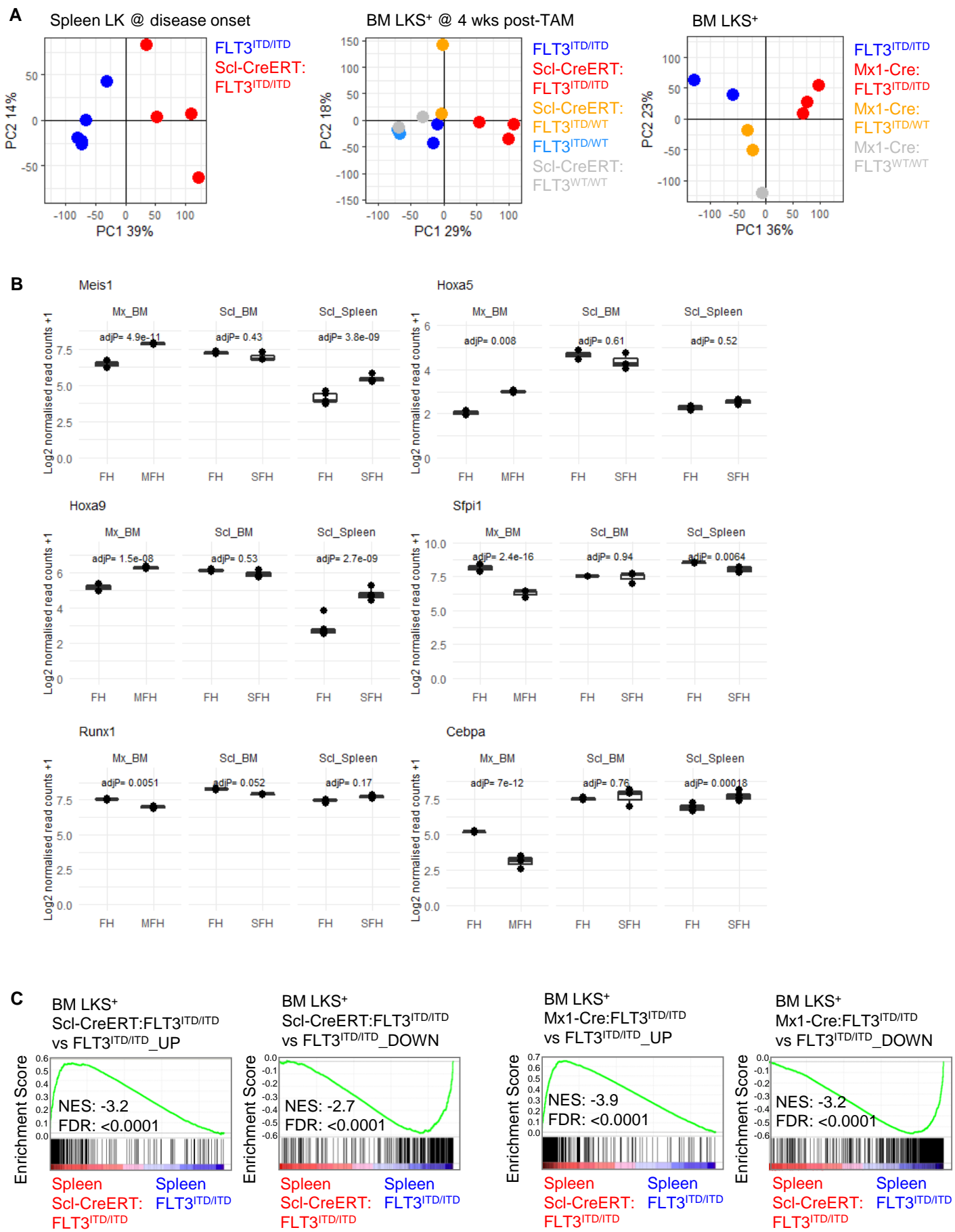

Supplement: Supplementary file 2 — Supplementary Fig. S2 [file 41375_2023_1832_MOESM2_ESM.pdf]

Supplementary Figure 3

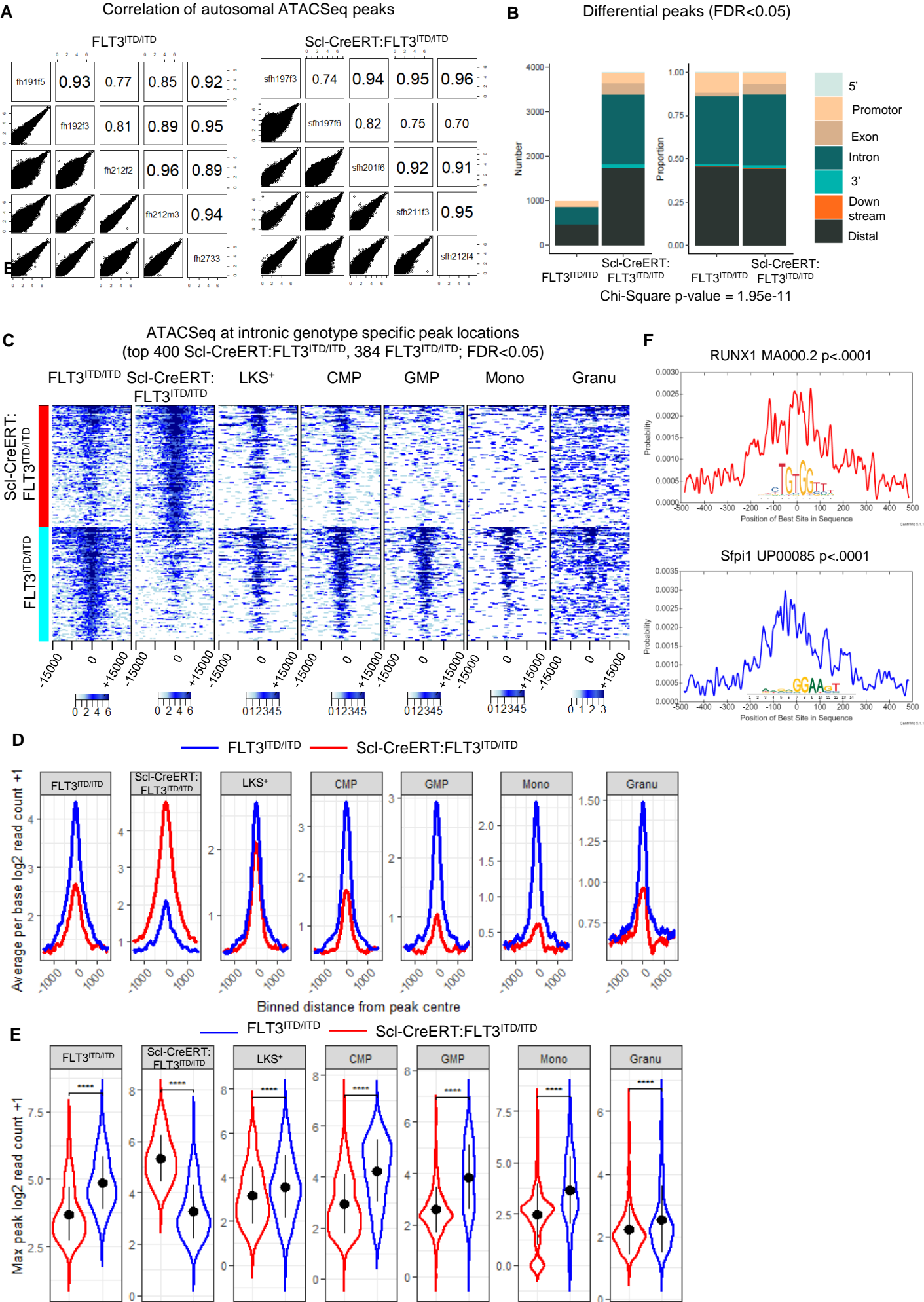

Supplement: Supplementary file 3 — Supplementary Fig. S3 [file 41375_2023_1832_MOESM3_ESM.pdf]

Supplementary Figure 4

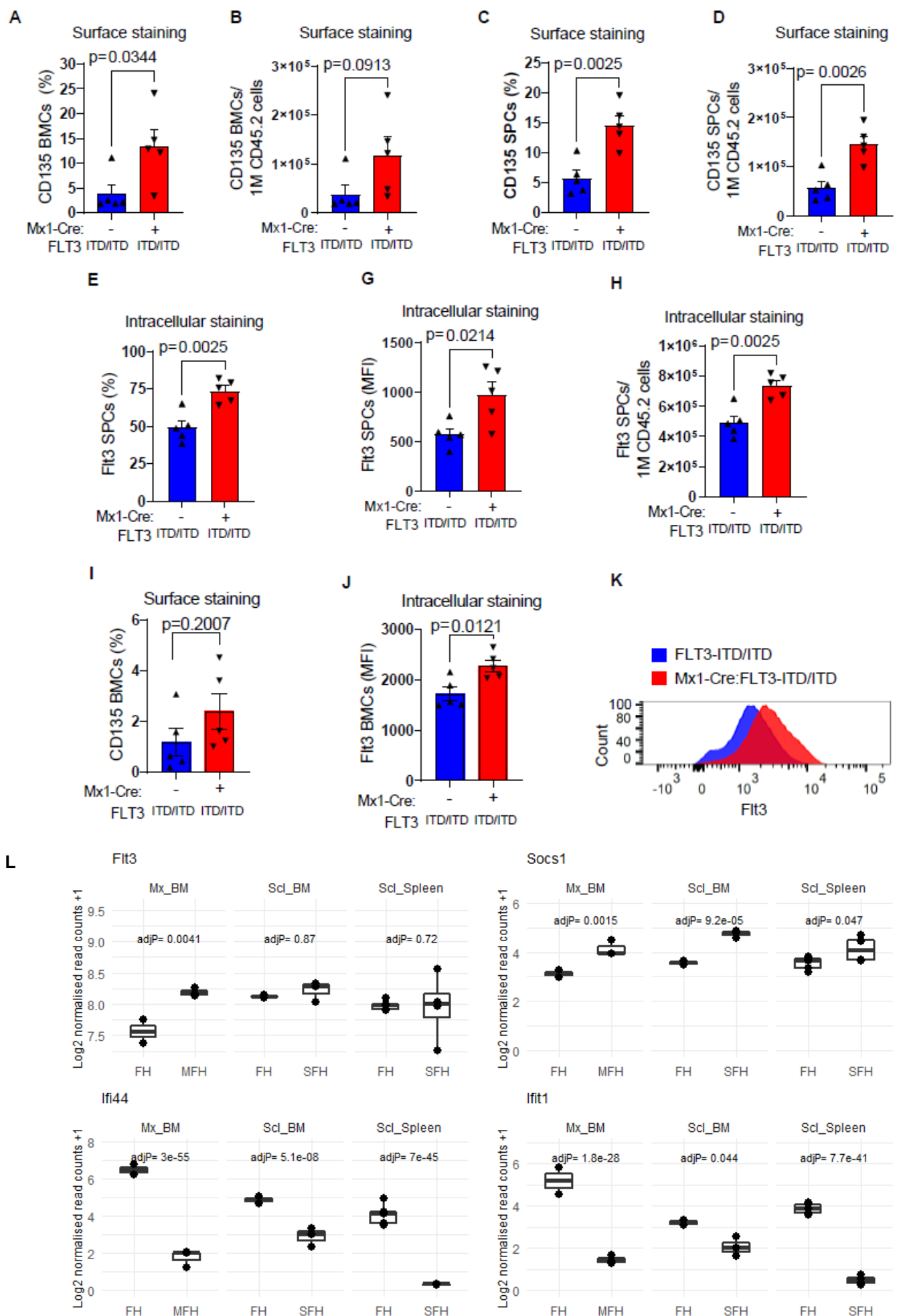

Supplement: Supplementary file 4 — Supplementary Fig. S4 [file 41375_2023_1832_MOESM4_ESM.pdf]
