# Supplementary material for: Cre recombinase expression cooperates with homozygous FLT3 internal tandem duplication knockin mouse model to induce acute myeloid leukemia
Source: Leukemia. 2023 Feb 4;37(4):741–50. doi: 10.1038/s41375-023-01832-0 (PMC10079527; doi:10.1038/s41375-023-01832-0)
Supplement: Supplementary file 5 — Supplementary Table S1 [file 41375_2023_1832_MOESM5_ESM.docx]

**Supplemental Table 1.** Used antibodies for flow cytometry analysis with respective targets, fluorophore, clone, manufacturer and ordering numbers

| **Target** | **Fluorophore** | **Clone** | **Manufacturer** | **Ordering number** |
| --- | --- | --- | --- | --- |
| Gr-1 (Ly-6G/Ly-6C) | APC | RB6-8C5 | Biolegend | 108412 |
| Mac-1 (CD11b) | PB | M1/70 | Biolegend | 101224 |
| Strepatvidin | PB | - | Biolegend | 405226 |
| Strepatvidin | APC-Cy7 | - | Biolegend | 405208 |
| Strepatvidin | BV421 |  | Biolegend | 405226 |
| FcyR (CD16/32) | PE-Cy7 | 93 | Biolegend | 405203 |
| Sca-1 (Ly-6A/E) | PE | D7 | Biolegend | 108108 |
| Sca-1 (Ly-6A/E) | FITC | D7 | Biolegend | 108106 |
| Sca-1 (Ly-6A/E) | PE-Cy7 | E13-161.7 | Biolegend | 122513 |
| Sca-1 (Ly-6A/E) | PE | E13-161.7 | Biolegend | 122507 |
| cKit (CD117) | APC | 2B8 | Biolegend | 105818 |
| CD150 (SLAM) | PerCP Cy5.5 | TC15-12F12.2 | Biolegend | 115922 |
| CD48 | APC-Cy7 | HM48-1 | Biolegend | 103431 |
| CD34 | FITC | RAM34 | BD Pharmingen | 553733 |
| CD135 | APC | A2F10 | Biolegend | 135309 |
| Sytox Blue Dead Cell Stain | PB | - | Thermo Fisher Scientific | S3857 |
| Gr-1 (Ly-6G/Ly-6C) | Biotin | RB6-8C5 | Biolegend | 108404 |
| B220 | Biotin | RA3-6B2 | Biolegend | 103204 |
| TERII9 | Biotin | TER-119 | Biolegend | 116204 |
| CD19 | Biotin | 6D5 | Biolegend | 115504 |
| CD3 | Biotin | 145-2C11 | Biolegend | 100304 |
| CD4 | Biotin | GK1.5 | Biolegend | 100404 |
| CD8a | Biotin | 53-6.7 | Biolegend | 121104 |
| IL-7 Rα | Biotin | SB/199 | Biolegend | 121104 |
| IL-7 Rα | Biotin | A7R34 | Biolegend | 135005 |
| Flt3 | - | 8F2 | Cell Signaling Technology | 3462 |
| DyLight^TM^ 649 Donkey anti-rabbit IgG | APC | - | Biolegend | 406406 |
